# Risk of Cerebral Palsy among the Offspring of Immigrants

**DOI:** 10.1371/journal.pone.0102275

**Published:** 2014-07-14

**Authors:** Joel G. Ray, Donald A. Redelmeier, Marcelo L. Urquia, Astrid Guttmann, Sarah D. McDonald, Marian J. Vermeulen

**Affiliations:** 1 Departments of Medicine and Obstetrics and Gynecology, St. Michael's Hospital and the Institute for Clinical Evaluative Sciences, University of Toronto, Toronto, Ontario, Canada; 2 Department of Medicine and Health Policy Management and Evaluation, Institute for Clinical Evaluative Sciences, University of Toronto, Toronto, Ontario, Canada; 3 Centre for Research on Inner City Health, St. Michael's Hospital, Institute for Clinical Evaluative Sciences, Toronto, Ontario, Canada; 4 Departments of Paediatrics and Health Policy, Management and Evaluation, The Hospital for Sick Children, Institute for Clinical Evaluative Sciences, University of Toronto, Toronto, Ontario, Canada; 5 Departments of Obstetrics & Gynecology, Diagnostic Imaging and Clinical Epidemiology and Biostatistics Division of Maternal-Fetal Medicine, McMaster University, Hamilton, Ontario, Canada; 6 Institute for Clinical Evaluative Sciences, University of Toronto, Toronto, Ontario, Canada; Oslo University Hospital, Ullevål, Norway

## Abstract

**Background:**

Cerebral palsy (CP) has a multifactorial etiology, and placental vascular disease may be one major risk factor. The risk of placental vascular disease may be lower among some immigrant groups. We studied the association between immigrant status and the risk of CP.

**Methods:**

We conducted a population-based retrospective cohort study of all singleton and twin livebirths in Ontario between 2002–2008, and who survived ≥28 days after birth. Each child was assessed for CP up to age 4 years, based on either a single inpatient or ≥2 outpatient pediatric diagnoses of CP. Relative to non-immigrants (n = 566,668), the risk of CP was assessed for all immigrants (n = 177,390), and further evaluated by World region of origin. Cox proportional hazard ratios (aHR) were adjusted for maternal age, income, diabetes mellitus, obesity, tobacco use, Caesarean delivery, year of delivery, physician visits, twin pregnancy, preterm delivery, as well as small- and large-for-gestational age birthweight.

**Results:**

There were 1346 cases of CP, with a lower rate among immigrants (1.45 per 1000) than non-immigrants (1.92 per 1000) (aHR 0.77, 95% confidence interval [CI] 0.67 to 0.88). Mothers from East Asia and the Pacific (aHR 0.54, 95% CI 0.39 to 0.77) and the Caribbean (aHR 0.58, 95% CI 0.37 to 0.93) were at a significantly lower risk of having a child with CP. Whether further adjusting for preeclampsia, gestational hypertension, placental abruption or placental infraction, or upon using a competing risk analysis that further accounted for stillbirth and neonatal death, these results did not change.

**Conclusions:**

Immigration and ethnicity appear to attenuate the risk of CP, and this effect is not fully explained by known risk factors.

## Background

Cerebral palsy (CP), describes a group of non-progressive disorders of movement and posture. Most cases are diagnosed before age 4 years. About 80% of cases are due to prenatal injury of the brain and only 10% are due to adverse events peripartum; most cases of CP occur in children with an apparently uncomplicated pregnancy [1]–[3]. The most recognized risk factors for CP are both low and high weight-for-gestation age (seen as a J-curve phenomenon) [4], as well as premature birth [5]; nonetheless, about half of all children who develop CP are born at term [5]. Predicting who is at highest risk of having a child with CP remains an international priority.

While the pathogenesis of CP may be elusive, *placental vascular disease* – a known cause of fetal growth restriction and prematurity [6]–[8] – appears to be a risk factor for CP and neurological impairment among low birthweight [9], term [10]–[12] and premature infants [13]. The “*maternal placental syndromes*”– preeclampsia, gestational hypertension, placental abruption & placental infarction – are often attributed to placental vascular disease [14]–[16], and seen in conjunction with intrauterine fetal growth restriction and preterm birth [17].

We previously observed a lower risk of *maternal placental syndromes* among recent immigrants to Canada, with a loss of the protective effect with increasing duration of residence [18]. Conversely, other studies noted a higher risk of serious preeclampsia and preterm delivery [19], [20], especially among immigrant women residing more than 10 years in Canada [20], and mothers originating from Latin America, the Caribbean and Sub-Saharan Africa [19].

While placental vascular disease likely predisposes to the *maternal placental syndromes*, prematurity and CP, there are conflicting data about the potential direction of that risk among immigrant women. Accordingly, we assessed the risk of CP among the children of immigrant vs. non-immigrant women, by immigrant region of origin, and by time since migration to Canada.

## Methods

### Study design

We completed a retrospective population-based cohort study, using pre-existing linked datasets at the Institute for Clinical Evaluative Sciences (ICES).

### Participants

We included all maternal-child pairs in the province of Ontario, with a singleton or twin obstetrical delivery at 23 weeks gestation or later, occurring within an Ontario hospital between April 1, 2002 and March 31, 2008. Since the child was the unit of analysis, a woman may have contributed more than one delivery in the study period. The time to the end of study was March 31, 2012, which permitted 4 years of potential follow-up for every child. We excluded mothers aged <14 years or >50 years and those who were not a resident of Ontario at the time of delivery. Neonatal deaths (0–27 days after birth) were excluded from the main model, but were included in a sensitivity analysis, described below.

### Exposures and outcomes

The main exposure of interest was maternal immigrant status prior to her delivery in Ontario. Immigrants were compared to non-immigrants – the latter comprising predominantly women born in Canada, and a smaller proportion of women who immigrated prior to January, 1985, who migrated to Ontario through another Canadian province or who could not be matched to a record in the immigration database. The secondary exposure of interest divided immigrant mothers by their World region of origin, according to the United Nations classification – a Western Nation or Europe, African/Caribbean, North African/Middle Eastern, Latin American, East Asian/Pacific and South Asian, and compared each World region to the non-immigrant group [19] (Table S1 in File S1).

The main study outcome was a diagnosis of CP in the 4-year period after each child's date of birth. A four year minimum was chosen since more than 95% of cases of CP are firmly diagnosed by that age [21], [22]. A diagnosis of CP was based on either of the following: i) Any inpatient hospitalization diagnosis of CP, using the International Statistical Classification of Diseases and Related Health Problems, 10^th^ Revision (ICD-10CA) diagnostic codes or ii) At least 2 outpatient outpatient diagnoses of CP≥14 days apart, each made by a licensed paediatrician on an Ontario Health Insurance Plan (OHIP) billings claims (Table S2 in File S1).

### Database sources

All analyses were conducted at ICES, where the existing databases are housed (see http://www.ices.on.ca/webpage.cfm?site_id=1&org_id=26&morg_id=0&gsec_id=5314&item_id=5322). All maternal, fetal and newborn infant hospitalizations and procedures were identified using the CIHI Discharge Abstract Database (DAD). The MOMBABY Dataset at ICES uses all DAD inpatient admission records of delivering mothers & their newborns from 2002 onward [7], [14], [18]–[20]. Mothers & their newborns are deterministically linked within the DAD. The MOMBABY dataset contains the unique encrypted healthcare number, age and sex of the participant, date of admission, and up to 25 diagnoses coded by ICD-10-CA.

Women who delivered a liveborn or stillbirth infant were linked to the Ontario portion of the federal Citizenship and Immigration Canada (CIC) Database, also housed at ICES [19], [23]. The CIC Database housed at ICES has records for every permanent legal immigrant to Ontario who arrived after 1984. While the father's country of birth is not known, we have shown a high concordance with the mother's World region of origin [24].

Since some conditions preceding and/or during pregnancy (e.g., maternal pre-pregnancy hypertension and diabetes mellitus) may be diagnosed as an outpatient, the OHIP Database was also used to identify covariates in the period up to 12 months before the index delivery hospitalization [7], [14]. This database contains records of all physician billing information for outpatient and inpatient services, including a service date and a single diagnosis. Maternal and child mortality were retrieved from the Registered Persons Database, which contains demographic information and the encrypted healthcare number for all individuals eligible for OHIP [7], [14]. Neighbourhood income quintile was defined according to postal code using Statistics Canada census data.

### Statistical analyses

#### Main model

All baseline participant characteristics were divided according to those 1) of the mother at date of admission for the index delivery hospitalization, 2) of the mother ≤12 months before, or during, the index delivery hospitalization, 3) of the mother during the index delivery hospitalization, 4) of the newborn in the index birth hospitalization, and 5) of the child in the index birth hospitalization and ≤12 months after birth. They are detailed in Table S2 in File S1.

In the main model, we used a time-to-event Cox proportional hazards model to compare the risk of CP between all immigrants and non-immigrants, the referent. The same was done when comparing specific immigrant World regions of origin to the non-immigrant reference group [19]. In the main analysis, the outcome event of CP was assessed starting from 28 days after birth (defined as time zero) up to 48 months after the date of birth. We censored if a child died from 28 days onward, up to aged 4 years.

A hazard ratio (HR) and 95% confidence interval (CI) was presented both unadjusted, as well as adjusted (aHR) for maternal age (continuous in years), parity, neighborhood income quintile, duration of residence in Canada (continuous in years), any pre-pregnancy or gestational diabetes mellitus, treated obesity or tobacco dependence, Caesarean delivery, fiscal year of delivery, number of physician visits between day 1 to 140 of pregnancy, a twin pregnancy, delivery before 32 or 37 weeks gestation, and small for gestational age birthweight under the 10^th^ percentile or large for gestational age birthweight above the 90^th^ percentile (Table S2 in File S1). The covariates were abstracted from DAD (for inpatient) and the OHIP Database (for outpatient) encounters ≤12 months prior to, or including, the index delivery hospitalization admission. They were chosen *a priori*, and were informed by previous studies on CP, placental vascular disease and/or pregnancy outcomes among immigrants [1]–[20].

#### Further analyses on the main model

We modified the main model by further adjusting for the presence of a maternal placental syndrome in the index delivery hospitalization, and reported both the modified aHR comparing immigrant mothers and non-immigrants, as well as the aHR for the presence vs. absence of a maternal placental syndrome in that model. For the main model, we stratified by income quintile (quintile 1 [low] or 5 [high]), maternal age (<35 or ≥35 years), parity (0 or ≥1), delivery (≥37 weeks, <37 weeks or <32 weeks), singleton vs. twin pregnancy, and number of features of the maternal placental syndrome – gestational hypertension, preeclampsia, placental abruption & placental infarction – in the index delivery hospitalization (0, 1 or ≥2).

To focus on placental vascular disease in the pathogenesis of CP, we re-ran the main model, but refined the study outcome to cases of CP without an explainable fetal/infant (e.g., congenital anomaly, prolapsed umbilical cord or birth trauma) or maternal (e.g., uterine rupture or chorioamnionitis) cause, as outlined in Table S2 in File S1.

### Sensitivity analyses of the competing risk of death

Infants at risk for CP are also at high risk of dying before they can potentially be diagnosed with CP [5]. Hence, we constructed a competing risk model for the endpoint of CP according to World region of origin, accounting for the competing event of death, using the cumulative incidence function curves for CP and death (“competing risk model #1”) [25]. The same covariates were used as in the main model.

Additionally, it is believed that CP and stillbirth share many common risk factors, including placental vascular pathology [26], [27]. Moreover, randomized clinical trials have considered stillbirth, infant death and CP within a primary composite outcome [28]. Thus, we also generated a competing risk model for the endpoint of CP according to World region of origin, accounting for the competing event of stillbirth and also for death after birth (competing risk model #2). In that analysis, we no longer excluded neonatal deaths before 28 days after birth.

#### Duration of residence and CP risk

The unadjusted rate (95% CI) of CP per 1000 infants alive ≥28 days after birth was plotted against the number of years between maternal immigration landing date and the index birth date, as well as among the non-immigrants.

All P-values were two-sided, at a significance level of 0.05. All statistical analyses were performed using SAS for UNIX, Version 9.2 (SAS Institute, Cary, NC), except for competing risk regression, which was done using the *stcrreg* command in STATA. The study was approved by the Ethics Review Board of Sunnybrook Health Sciences Centre, Toronto, Ontario, Canada.

## Results

For the main model, we initially identified 805,215 newborns, of whom we further excluded 61,159 for the following reasons: invalid health card number (n = 2,015), duplicates (n = 642), non-Ontario resident (n = 47,485), age <14 or >50 years (n = 129), triplet or higher order pregnancy (n = 1,449), stillbirth (n = 709), newborn gestational age <23 weeks at birth (n = 239), birthweight <500 grams (n = 100) or neonatal death <28 days (n = 2,719), or immigrant landing date or country of origin unknown (n = 5,672). Hence, there were 744,058 newborns in the main cohort (92.9%) (Figure S1 in File S2).

There were 566,668 and 177,390 infants born to non-immigrant and immigrant mothers, respectively (Table 1). Of all infants born to immigrant mothers from various World regions nearly 32% were from South Asian countries and 22% from East Asian and the Pacific countries (Table 1). The characteristics of the mothers, their delivery and their newborns are listed in Table 1. Non-immigrants (45.8%) were more likely to be nulliparous than mothers from Sub-Saharan Africa (30.9%), and were more likely than all immigrant groups to dwell in a high-income area. The rate of diabetes mellitus was lowest among non-immigrants (5.7%) and immigrants from Western Nations and Europe (5.6%) in contrast to other immigrant groups (Table 1). Twin pregnancy was most prevalent among non-immigrant pregnancies, as was the *maternal placental syndrome* (7.1%), with the exception of mothers originating from the Caribbean (7.7%). The prevalence of some CP risk factors, such as chorioamnionitis, preterm birth, or small for gestational age birthweight, did not differ appreciably between immigrants and non-immigrants, while others, including complications of labour and delivery or noxious influences transmitted via placenta or breast milk, did (Table 1).

**Table 1:** Characteristics of mothers and infants in the main cohort.

			World region of origin among immigrant mothers						
Characteristic		*Non-immigrants*	Sub-Saharan Africa	South Asia	Caribbean	Hispanic America	Middle East & North Africa	Western Nations & Europe†	East Asia& Pacific
		*(n = 566,668)*	(n = 12,717)	(n = 56,316)	(n = 10,899)	(n = 13,417)	(n = 17,222)	(n = 27,967)	(n = 38,852)
*Of the mother at date of admission for the index delivery hospitalization*									
Mean (SD maternal age, years		29.7 (5.6)	31.0 (5.4)	29.1 (4.6)	29.2 (6.4)	29.8 (5.7)	30.0 (5.4)	30.8(5.2)	32.1(5.0)
Parity	*0*	259,423 (45.8)	3,929 (30.9)	23,924 (42.5)	4,355 (40.0)	5,770 (43.0)	6,838 (39.7)	13,260 (47.4)	18,910 (48.7)
	*1*	201,843 (35.6)	3,610 (28.4)	21,303 (37.8)	3,623 (33.2)	4,818 (35.9)	5,694 (33.1)	10,354 (37.0)	15,523 (40.0)
	*2–3*	94,449 (16.7)	3,756 (29.5)	10,210 (18.1)	2,539 (23.3)	2,485 (18.5)	3,982 (23.1)	3,871 (13.8)	4,238 (10.9)
	*4+*	10,934 (1.9)	1,421 (11.2)	876 (1.6)	382 (3.5)	344 (2.6)	707 (4.1)	481 (1.7)	181 (0.5)
Income quintile (Q)	*Q1*	101,721 (18.0)	7,034 (55.3)	22,265 (39.5)	4,604 (42.2)	4,388 (32.7)	6,447 (37.4)	5,980 (21.4)	11,983 (30.8)
	*Q2*	107,140 (18.9)	2,334 (18.4)	14,325 (25.4)	2,574 (23.6)	3,266 (24.3)	3,415 (19.8)	5,398 (19.3)	10,106 (26.0)
	*Q3*	118,112 (20.8)	1,495 (11.8)	10,651 (18.9)	2,015 (18.5)	2,628 (19.6)	2,910 (16.9)	5,634 (20.2)	7,613 (19.6)
	*Q4*	126,559 (22.3)	1,142 (9.0)	6,406 (11.4)	1,149 (10.5)	1,943 (14.5)	2,806 (16.3)	6,049 (21.6)	5,690 (14.7)
	*Q5*	110,198 (19.5)	685 (5.4)	2,632 (4.7)	550 (5.1)	1,183 (8.8)	1,617 (9.4)	4,884 (17.5)	3,403 (8.8)
No. physician visits from day 1 to day 140 of pregnancy		7.3 (4.0)	7.8 (4.6)	8.3 (4.5)	8.3 (4.4)	7.9 (4.2)	7.6 (4.7)	7.5 (4.0)	7.5 (3.8)
*Of the mother ≤12 months before, or during, the index delivery hospitalization*									
Pre-pregnancy or gestational diabetes mellitus		32,061 (5.7)	1,294 (10.2)	8,096 (14.4)	1,050 (9.6)	1,239 (9.2)	1,391 (8.1)	1,574 (5.6)	4,181 (10.8)
Obesity		7,350 (1.3)	177 (1.4)	514 (0.9)	240 (2.2)	198 (1.5)	200 (1.2)	220 (0.79)	144 (0.37)
Tobacco use disorder		1,470 (0.3)	≤5	≤5	8 (0.07)	10 (0.07)	6 (0.03)	42 (0.2)	≤5
*Of the mother during the index delivery hospitalization*									
Cesarean delivery		155,916 (27.5)	4,021 (31.6)	15,496 (27.5)	3,088 (28.3)	3,999 (29.8)	4,329 (25.1)	6,814 (24.4)	10,764 (27.7)
Twin pregnancy		19,220 (3.4)	376 (3.0)	1,241 (2.2)	281 (2.6)	337 (2.5)	548 (3.2)	875 (3.1)	714 (1.8)
Maternal placental syndrome*		39,938 (7.1)	735 (5.8)	2,447 (4.3)	841 (7.7)	688 (5.1)	644 (3.7)	1,498 (5.4)	1,704 (4.4)
Intrauterine hypoxia and birth asphyxia		37 (0.01)	0 (0.0)	0 (0.0)	0 (0.0)	0 (0.0)	0 (0.0)	≤5	0 (0.0)
Uterine rupture		520 (0.09)	21 (0.2)	39 (0.07)	≤5	≤5	10 (0.06)	14 (0.05)	12 (0.03)
Umbilical cord prolapse or vasa previa		71,512 (12.6)	1,908 (15.0)	7,884 (14.0)	1,551 (14.2)	1,721 (12.8)	2,684 (15.6)	4,003 (14.3)	4,658 (12.0)
Amniotic fluid embolism		22 (0.0)	0 (0.0)	≤5	≤5	≤5	≤5	0 (0.0)	≤5
Fetal-maternal hemorrhage		217 (0.04)	≤5	12 (0.02)	≤5	≤5	8 (0.05)	7 (0.03)	6 (0.02)
Chorioamnionitis		4,094 (0.7)	112 (0.9)	552 (1.0)	134 (1.2)	124 (0.9)	104 (0.6)	216 (0.8)	508 (1.3)
Median (IQR) number of years of residence		–	6 (3–11)	4 (2–7)	10 (6–14)	8 (3–13)	4 (2–8)	7 (3–13)	5 (2–9)
*Of the newborn in the index birth hospitalization*									
No. (%) female		273,652 (48.3)	6,112 (48.1)	27,054 (48.0)	5,373 (49.3)	6,430 (47.9)	8,322 (48.3)	13,283 (47.5)	18,747 (48.3)
Mean (SD) gestational age at delivery, weeks		38.9 (1.9)	38.9 (2.0)	38.8 (1.8)	38.5 (2.3)	38.8 (1.9)	39.0 (1.7)	39.0 (1.8)	38.8 (1.7)
Preterm birth <32 weeks gestation		5,492 (01.0)	149 (1.2)	491 (0.9)	244 (2.2)	132 (1.0)	124 (0.7)	220 (0.8)	280 (0.7)
Preterm birth 32 to <37 weeks gestation		39,050 (6.9)	723 (5.7)	3,293 (5.9)	877 (8.1)	852 (6.4)	906 (5.3)	1,605 (5.7)	2,143 (5.5)
Mean (SD) birthweight, grams		3,411 (584)	3,336 (586)	3,214 (534)	3,208 (619)	3,294 (561)	3,350 (528)	3,429 (555)	3,268 (504)
Small for gestational age birthweight									
<10^th^ percentile		61,446 (10.8)	1,864 (14.7)	11,925 (21.2)	1,944 (17.8)	2,127 (15.9)	2,331 (13.5)	2,886 (10.3)	6,215 (16.0)
Large for gestational age birthweight									
>90^th^ percentile		52,787 (9.3)	868 (6.8)	2,192 (3.9)	613 (5.6)	756 (5.6)	991 (5.8)	2,269 (8.1)	1,693 (4.4)
Respiratory distress syndrome		40,230 (7.1)	885 (7.0)	4,076 (7.2)	945 (8.7)	782 (5.8)	913 (5.3)	1,665 (6.0)	1,731 (4.5)
Necrotizing enterocolitis		324 (0.06)	7 (0.06)	29 (0.05)	8 (0.07)	≤5	≤5	9 (0.03)	11 (0.03)
Periventricular leukomalacia or intraventricular hemorrhage		1,291 (0.2)	38 (0.3)	119 (0.2)	48 (0.4)	25 (0.2)	24 (0.1)	61 (0.2)	73 (0.2)
Retinopathy of prematurity		924 (0.2)	33 (0.3)	87 (0.2)	36 (0.3)	24 (0.2)	22 (0.1)	51 (0.2)	38 (0.1)
Placenta praevia or umbilical cord problem		2,926 (0.5)	32 (0.3)	213 (0.4)	33 (0.3)	40 (0.3)	45 (0.3)	82 (0.3)	110 (0.3)
Infections specific to the perinatal period		4,260 (0.8)	122 (1.0)	394 (0.7)	106 (1.0)	89 (0.7)	100 (0.6)	208 (0.7)	204 (0.5)
Chorioamnionitis		332 (0.06)	12 (0.09)	58 (0.1)	18 (0.2)	13 (0.1)	8 (0.05)	12 (0.04)	37 (0.1)
Kernicterus		6 (0.0)	0 (0.0)	≤5	0 (0.0)	0 (0.0)	≤5	0 (0.0)	≤5
Fetal and neonatal hemorrhage		1,652 (0.3)	22 (0.2)	70 (0.1)	14 (0.1)	14 (0.1)	19 (0.1)	56 (0.2)	88 (0.2)
Fetus and newborn affected by other complications of labour and delivery		5,564 (1.0)	76 (0.6)	307 (0.6)	57 (0.5)	74 (0.6)	76 (0.4)	121 (0.4)	206 (0.5)
Noxious influences transmitted via placenta or breast milk		2,001 (0.4)	15 (0.1)	33 (0.06)	19 (0.2)	18 (0.1)	18 (0.1)	37 (0.1)	18 (0.05)
*Of the child in the index birth hospitalization and ≤12 months after birth*									
Any congenital or chromosomal anomaly		15,647 (2.8)	383 (3.0)	1,273 (2.3)	328 (3.0)	275 (2.1)	381 (2.2)	666 (2.4)	824 (2.1)

Data are for singleton or twin liveborn infant who survived 28 days or more after birth, according to World region of origin. All data are presented as a number (percent) unless otherwise indicated. Any cell size “≤5” is suppressed.

*Preeclampsia or eclampsia, gestational hypertension, placental abruption and/or placental infarction.

† Comprises all of Europe, the UK, Wales, Scotland and Ireland, Australia and New Zealand, and the US.

IQR Interquartile range.

### Main model

**Figure 1:**
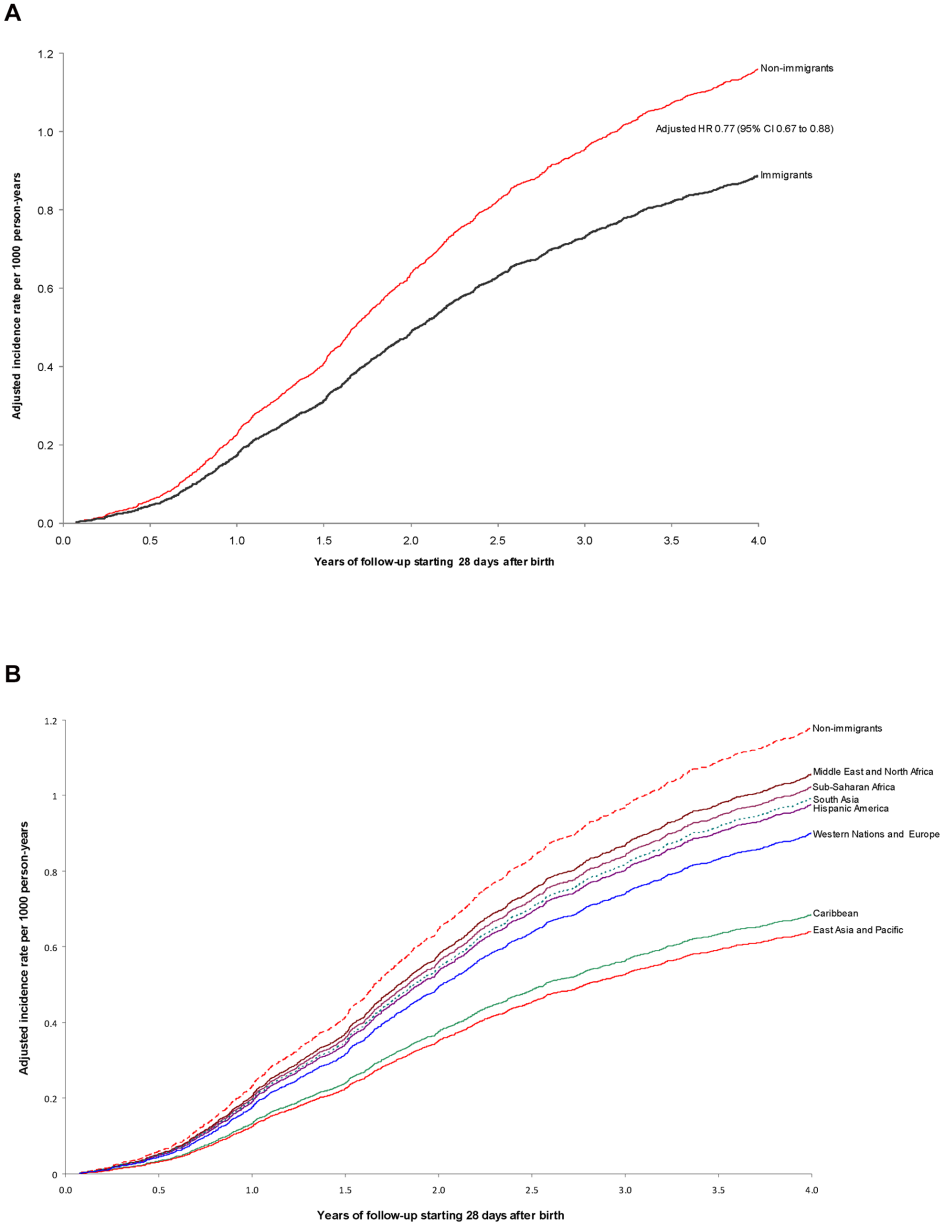
Risk of cerebral palsy (CP) comparing immigrants to non-immigrants (Figure 1a), as well as immigrants by World region of origin to non-immigrants (Figure 1b). All data were analyzed starting 28 days after birth, and adjusted for maternal age, parity, income quintile, any diabetes mellitus, obesity, tobacco use, Caesarean delivery, fiscal year of delivery, number of physician visits between day 1 and day 140 of pregnancy, twin pregnancy, preterm birth before 32 weeks, preterm birth from 33 to 37 weeks, small for gestational age birthweight under the 10th percentile, and large for gestational age birthweight over 90th percentile.

There were 1346 cases of CP among 744,058 infants (1.81 per 1000) surviving 28 days or more after birth. The rate of CP was lower among the children of immigrants (1.45 per 1000 [95% CI 1.28 to 1.64) than non-immigrants (1.92 per 1000 [95% CI 1.81 to 2.04]), equivalent to a crude HR of 0.75 (95% CI 0.66 to 0.86) and an aHR of 0.77 (95% CI 0.67 to 0.88) (Table 2, Figure 1a). When further broken down by maternal World region of origin, only those from East Asia and Pacific (aHR 0.54, 95% CI 0.39 to 0.77) and the Caribbean (aHR 0.58, 95% CI 0.37 to 0.93) had a significantly lower risk of having a child with CP (Table 2, Figure 1b).

**Table 2:** Main model analyses examining the risk of childhood cerebral palsy by maternal immigration status (upper) and by immigrant World region of origin (lower), each compared to non-immigrants.

		Outcome of cerebral palsy up to age 4 years		
			Hazard ratio (95% CI)	
Analysis	Mother's World region of origin	Number of events(rate per 1000 [95% CI])	Unadjusted	Adjusted*
***Comparing immigrant vs. non-immigrants***	Non-immigrants (n = 566,668)	1089 (1.92 [1.81 to 2.04])	*1.00 (referent)*	*1.00 (referent)*
	Immigrant (n = 177,390)	257 (1.45 [1.28 to 1.64])	0.75 (0.66 to 0.86)	0.77 (0.67 to 0.88)
***Comparing immigrants by their World region vs. non-immigrants***	Non-immigrants (n = 566,668)	1089 (1.92 [1.81 to 2.04])	*1.00 (referent)*	*1.00 (referent)*
	Sub-Saharan Africa (n = 12,717)	23 (1.81 [1.21 to 2.71])	0.94 (0.62 to 1.42)	0.87 (0.57 to 1.32)
	South Asia (n = 56,316)	94 (1.67 [1.37 to 2.04])	0.87 (0.70 to 1.07)	0.84 (0.68 to 1.05)
	Caribbean (n = 10,899)	18 (1.65 [1.05 to 2.61])	0.86 (0.54 to 1.37)	0.58 (0.37 to 0.93)
	Hispanic America (n = 13,417)	22 (1.64 [1.09 to 2.48])	0.85 (0.56 to 1.30)	0.83 (0.54 to 1.27)
	Middle East and North Africa (n = 17,222)	28 (1.63 [1.13 to 2.35])	0.85 (0.58 to 1.23)	0.90 (0.61 to 1.32)
	Western Nations and Europe (n = 27,967)	37 (1.32 [0.96 to 1.82])	0.70 (0.50 to 0.96)	0.77 (0.55 to 1.06)
	East Asia and Pacific (n = 38,852)	35 (0.90 [0.65 to 1.25])	0.47 (0.34 to 0.66)	0.54 (0.39 to 0.77)

The period of observation starts at 28 days after birth and continues until age 4 years.

*Adjusted for maternal age, parity, neighbourhood income quintile, any pre-pregnancy or gestational diabetes mellitus, obesity, tobacco use, Caesarean delivery, fiscal year of delivery, number of physician visits between day 1 and day 140 of pregnancy, twin pregnancy, preterm birth before 32 weeks, preterm birth from 33 to 37 weeks, small for gestational age birthweight under the 10th percentile, and large for gestational age birthweight over the 90th percentile.

CI confidence interval.

### Further analyses on the main model

The addition of a maternal placental syndrome to the main model did not further alter the aHR (Table S3 in File S1). In the latter modified model, the presence vs. absence of a maternal placental syndrome was associated with a slightly higher risk of CP (aHR 1.16, 95% CI 1.00 to 1.35).

**Figure 2:**
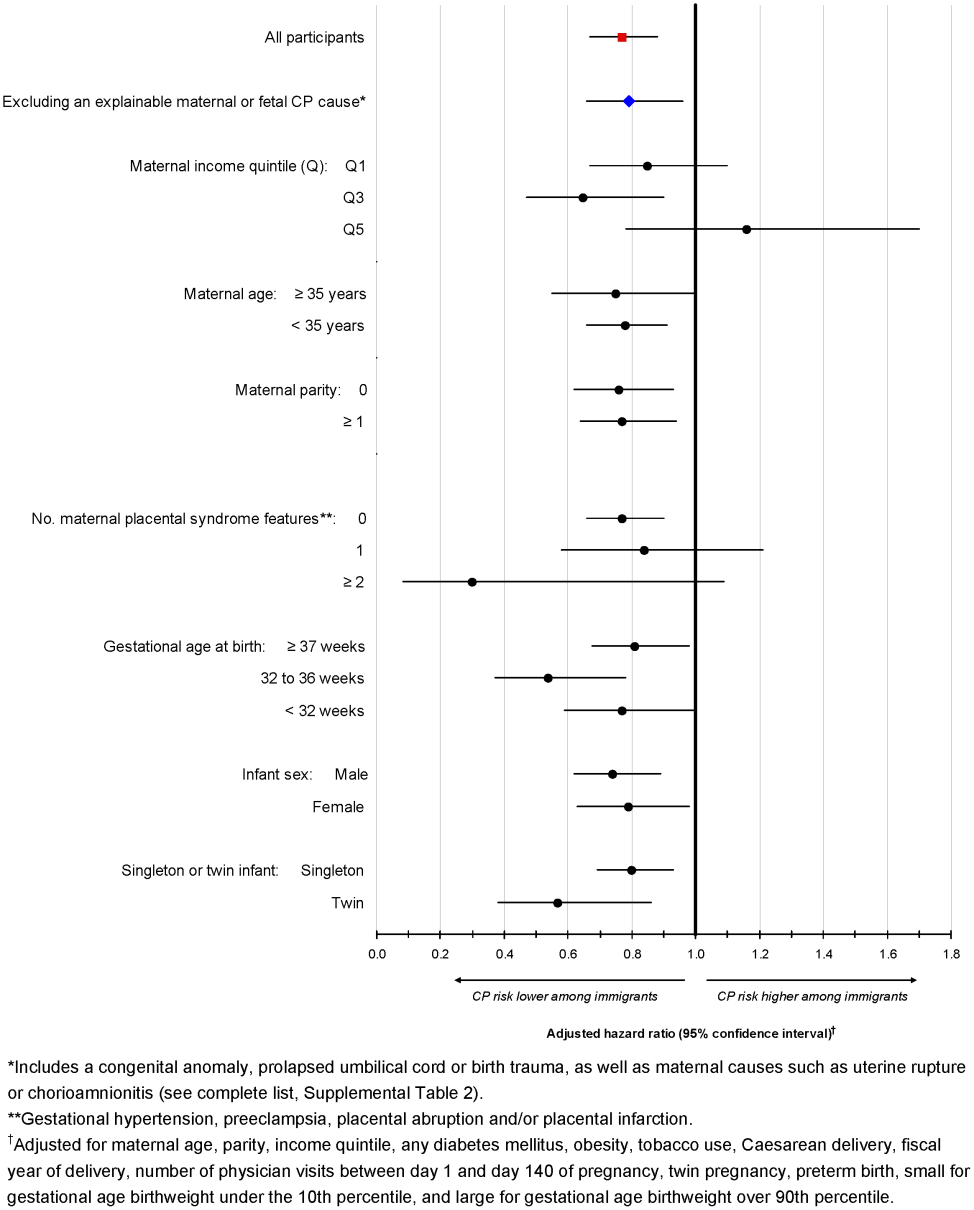
Risk of cerebral palsy (CP) among all participants (red square), those without a potential explainable cause of CP (blue diamond), and all participants stratified by maternal or newborn characteristics (black circles). All data were analyzed using the main Cox proportional hazard model.

In the main model, comparing immigrants to non-immigrants, the exclusion of those with an explainable cause of CP did not change the main findings (Figure 2). Similarly, among all children, stratifying by maternal or newborn factors did not appreciably change the results, including a twin pregnancy or preterm birth (Figure 2). The exception was among those residing in a high-income level area, wherein immigrants were no longer at a lower risk of having a child with CP. Among those mothers with 2 or more *maternal placental syndrome* elements, the adjusted risk of childhood CP was non-significantly lower among immigrants (HR 0.30, 95% CI 0.08 to 1.09).

### Competing risk models

In competing risk model #1, where infant death from 28 days onward was treated as a competing risk, the results were similar to those in the main model (Table 3, upper). In competing risk model #2, an additional 8454 stillbirths or neonatal deaths under 28 days were included in the non-immigrant group (revised n = 575,122) and another 2497 in the immigrant group (revised n = 179,887), contributing another 20 and 3 cases of CP, respectively. Again, the risk of CP was lower among women from East Asia and the Pacific, and the Caribbean (Table 3, lower).

**Table 3:** Competing risk models for cerebral palsy accounting for death from 28 days after birth up to age 4 years (upper), as well as for stillbirth or death from the first day of birth up to age 4 years (lower), in association with maternal World region of origin.

			Hazard ratio (95% CI)	
Competing risk model	Analysis	Mother's World region of origin	Unadjusted	Adjusted*
**Model #1: Competing risk of death from 28 days after birth up to age 4 years**	***Comparing immigrant vs. non-immigrants***	Non-immigrants (n = 566,668)	*1.00 (referent)*	*1.00 (referent)*
		Immigrant (n = 177,390)	0.74 (0.64 to 0.86)	0.78 (0.67 to 0.90
	***Comparing immigrants by their World region vs. non-immigrants***	Non-immigrants (n = 566,668)	*1.00 (referent)*	*1.00 (referent)*
		Sub-Saharan Africa (n = 12,717)	0.87 (0.56 to 1.35)	0.79 (0.50 to 1.23)
		South Asia (n = 56,316)	0.86 (0.69 to 1.07)	0.84 (0.67 to 1.05)
		Caribbean (n = 10,899)	0.86 (0.53 to 1.39)	0.59 (0.36 to 0.96)
		Hispanic America (n = 13,417)	0.82 (0.53 to 1.28)	0.79 (0.51 to 1.23)
		Middle East and North Africa (n = 17,222)	0.90 (0.62 to 1.30)	0.96 (0.66 to 1.40)
		Western Nations and Europe (n = 27,967)	0.69 (0.49 to 0.97)	0.78 (0.55 to 1.09)
		East Asia and Pacific (n = 38,852)	0.45 (0.32 to 0.65)	0.53 (0.37 to 0.76)
**Model #2: Competing risk of stillbirth or death from the first day of birth up to age 4 years** **	***Comparing immigrant vs. non-immigrants***	Non-immigrants (n = 575,122)	*1.00 (referent)*	*1.00 (referent)*
		Immigrant (n = 179,887)	0.74 (0.64 to 0.86)	0.77 (0.66 to 0.89)
	***Comparing immigrants by their World region vs. non-immigrants***	Non-immigrants (n = 575,122)	*1.00 (referent)*	*1.00 (referent)*
		Sub-Saharan Africa (n = 12,981)	0.86 (0.55 to 1.33)	0.75 (0.48 to 1.18)
		South Asia (n = 57,168)	0.86 (0.69 to 1.06)	0.82 (0.65 to 1.03)
		Caribbean (n = 11,176)	0.85 (0.52 to 1.37)	0.56 (0.35 to 0.91)
		Hispanic America (n = 13,640)	0.82 (0.52 to 1.27)	0.77 (0.50 to 1.21)
		Middle East and North Africa (n = 17,433)	0.89 (0.61 to 1.30)	0.94 (0.65 to 1.38)
		Western Nations and Europe (n = 28,274)	0.71 (0.51 to 0.99)	0.78 (0.56 to 1.09)
		East Asia and Pacific (n = 39,215)	0.47 (0.33 to 0.66)	0.53 (0.37 to 0.75)

*Adjusted for maternal age, parity, neighbourhood income quintile, any pre-pregnancy or gestational diabetes mellitus, obesity, tobacco use, Caesarean delivery, fiscal year of delivery, number of physician visits between day 1 and day 140 of pregnancy, twin pregnancy, preterm birth before 32 weeks, preterm birth from 33 to 37 weeks, small for gestational age birthweight under the 10th percentile, and large for gestational age birthweight over the 90th percentile.

**The number of deliveries is higher than in the other analyses, as stillbirths and neonatal deaths <28 days after birth were included in this competing risk model.

CI confidence interval.

### Duration of residence and CP risk

**Figure 3:**
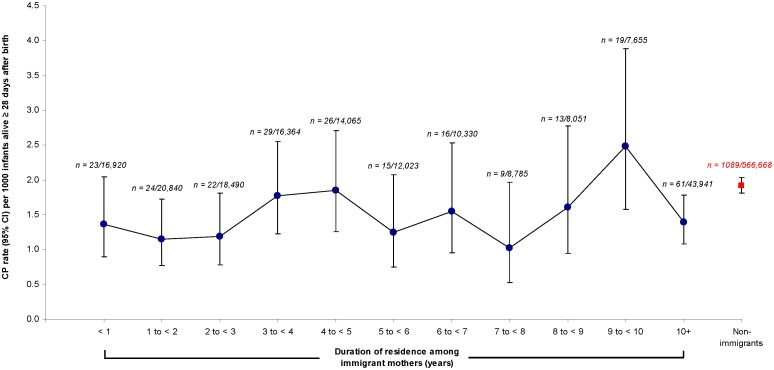
Unadjusted rate of cerebral palsy (CP) per 1000 singleton or twin infants, assessed from 28 days after birth up to 4 years of age. Data are shown according to duration of residence among immigrant mothers (black dots) and also among non-immigrant mothers (red square). The number of cases of CP cases over the number of infants are shown for each interval (e.g., *“n = 23/16,920”*).

Among immigrant women, there was minimal modulation in the risk of CP with maternal duration of residence prior to the index obstetrical delivery (Figure 3).

## Discussion

Among nearly 750,000 newborns, those born to non-immigrant women – especially among women from the Caribbean and East Asia – were at significantly lower risk of CP than those of non-immigrant women. This effect remained across various sub-group and competing risk analyses. The risk of CP did not vary despite accounting for explainable causes of CP or by duration of residence in Canada.

### Potential study limitations and strengths

Compared to other chronic conditions, CP is less well captured in some pediatric ambulatory databases [29]. We attempted to maximize the specificity of a CP diagnosis based on either a hospitalization or ≥2 outpatient visits to a pediatrician ≥14 days apart, and we required a follow-up to age 4 years. In doing so, our overall rate of CP was similar to that in other studies [5], [21], [22], [28]. It is unlikely that CP was more commonly missed in the children of immigrant women, since all were enrolled in a universal healthcare system and follow-up was to age 4 years. While we did not account for the ethnicity of the non-immigrant women, the majority would be of British and European ancestry [30].

We had a novel opportunity to link immigrant status to other health databases. We were able to consider both important covariates and to model the competing risk of death. Together, we systematically and precisely evaluated the relation between maternal immigration (and immigrant-defined ethnicity) and CP risk. However, obesity and tobacco use were each based on diagnostic codes from hospital and physicians' service claims, so we likely missed many women who did not receive formal counselling. Documentation of child health events or exposures after the neonatal period was also limited. Such unmeasured potential confounders may have biased our estimates of the associated risk between immigration and CP.

### Mechanisms

The pathogenesis of CP is multifactorial. Factors contributing to fetal brain injury may be acute (i.e., within hours) or chronic (i.e., over days or weeks), and continuous or intermittent [1]. In the current study, we ran a sensitivity analysis that excluded known risk factors for CP, and the effect size did not change. When preeclampsia, preterm delivery and SGA occur concomitantly, placental vasculopathy is likely to be present [13], [31]. In the current study, we did not have actual placental pathology reports; rather, *maternal placental syndromes* were used as an indirect measure of placental vascular disease [6]–[8], [16], [17]. In two other studies, preeclampsia was associated with an increased risk of CP among infants born ≥37 weeks gestation (OR 5.1, 95% CI 2.2 to 12.0) [32], especially when severe SGA was co-present [33], [34]. However, preeclampsia conveyed a lower risk of CP among those born ≤32 weeks (OR 0.39, 95% CI 0.15 to 0.93) [32], especially in the absence of SGA (OR 0.45, 95% CI 0.25 to 0.80) [34]. There are at least two reasonable explanations for these contrasting findings. First, the aforementioned studies excluded stillbirths [32], [34] and infants who died in the first week of life [34]; yet, cerebral damage, most likely in infants born very preterm to mothers with preeclampsia, are particularly likely to die. It is for this reason that we ran a competing risk analysis that considered both stillbirth and neonatal death, which did not alter our findings.

A second explanation may be that the pathogenesis of CP in the very preterm term infant differs from that in the term infant, wherein the former is due to an acute insult, while the latter is more chronic and indolent. However, preeclampsia is not the only *maternal placental syndrome* event associated with CP; so too are placental abruption [35] and placental infarction in the absence of preeclampsia [36]. Herein, the presence of a *maternal placental syndrome* did not alter the risk of CP in relation to immigrant status, and was of marginal significance in and of itself, nor did adjustment for preterm birth or abnormal birthweight. A direct assessment of placental pathology and its relation to *maternal placental syndrome*, fetal growth restriction and preterm delivery, would optimally shed light on whether certain immigrant groups are at lower risk of placental vascular disease.

The “healthy immigrant effect” may partly explain why the offspring of immigrants are at lower risk of CP. This may partly be a function of Canada's immigration policy, which mostly admits persons who are skilled and educated. For example, recent immigrants have lower rates of obesity and chronic hypertension in pregnancy [18] and in non-pregnant adulthood [27]–[39]. Since obesity, chronic hypertension and diabetes mellitus each are independent risk factors for placental dysfunction [7] and CP [40], it is plausible that the risk of CP is lower in some immigrant groups.

### Study implications

Does maternal immigrant status or maternal ethnicity predict CP risk? Among a cohort of 1588 preterm neonates included in the Beneficial Effects of Antenatal Magnesium Sulfate (BEAM) study, the authors evaluated whether use of individualized fetal growth standards, which include maternal ethnicity, better identifies SGA infants at risk of CP or death [41]. While maternal ethnicity was only defined as “Black” (45%), “White” (38%) or “Hispanic” (15%), and immigrant status was not assessed, the area under the curve for predicting CP or death was slightly better with the individualized growth standard (0.59, 95% CI 0.54 to 0.64) than the population standard (0.55, 95% CI 0.49 to 0.60) [41]. In a 10-year retrospective cohort study using California birth records, relative to Whites, the crude relative risk of CP was slightly higher among Blacks (1.29, 95% CI 1.19 to 1.39), and was lower among Asians (0.80, 95% CI 0.74 to 0.87) [42]. The “Asian” group comprised both South Asians and East Asians, and again, immigrant status was unknown. Moreover, after controlling for weight or gestational age at birth, Black race was actually associated with a lower risk of CP, suggesting that both factors explained the increased risk of CP in Black infants. In our study, we also observed a further attenuation of the risk of CP among the infants of mothers from either Sub-Saharan Africa or the Caribbean after adjusting for prematurity and extremes of birthweight (Table 2).

Ethnicity/race and immigration status appear to be risk factors for CP. Using as detailed a description for each, as is feasible, can further elucidate the likelihood that a woman's pregnancy may result in a child affected by CP.

## Supporting Information

File S1 **Supporting Tables S1, S2, and S3. Table S1. List of countries used to define World region of origin among the immigrant women included in the study. Table S2. Diagnostic and procedural codes used to identify the cohort, comorbidity and outcome features. Table S3. Modified main model, also adjusting for the presence of a maternal placental syndrome during the index delivery hospitalization.**
(DOC)

Figure S1 **Participant selection for the main cohort model**
(TIF)
