# Supplementary material for: Risk of Cerebral Palsy among the Offspring of Immigrants
Source: PLoS One. 2014 Jul 14;9(7):e102275. doi: 10.1371/journal.pone.0102275 (PMC4096602; doi:10.1371/journal.pone.0102275)
Supplement: File S1 — Supporting Tables S1, S2, and S3. Table S1. List of countries used to define World region of origin among the immigrant women included in the study. Table S2. Diagnostic and procedural codes used to identify the cohort, comorbidity and outcome features. Table S3. Modified main model, also adjusting for the presence of a maternal placental syndrome during the index delivery hospitalization. (DOC) [file pone.0102275.s001.doc]

**Table S1. List of countries used to define World region of origin among the immigrant women included in the study**

| **Western Nations and Europe**: Albania, Andorra, Australia, Austria, Belarus, Belgium, Bermuda, Bosnia and Herzegovina, Bulgaria, Croatia, Czech Republic, Denmark, Estonia, Finland, Former Czechoslovakia, Former USSR, Former Yugoslavia, Falkland Islands, France, Germany, Greece, Greenland, Hungary, Iceland, Ireland, Italy, Kosovo, Latvia, Liechtenstein, Lithuania, Luxembourg, Macedonia, Malta, Moldova, Monaco, Montenegro, Netherlands, New Zealand, Norway, Pitcairn Island, Poland, Portugal, Romania, Russian Federation, San Marino, Serbia, Serbia and Montenegro, Slovakia, Slovenia, Southern Antarctic Territories, Spain, St Helena, St Pierre and Miquelon, Sweden, Switzerland, Ukraine, United Kingdom, United States, Vatican City State. |
| --- |
| **Hispanic America**: Argentina, Bolivia, Brazil, Chile, Colombia, Costa Rica, Cuba, Dominican Republic, Ecuador, El Salvador, French Guiana, Guatemala, Guyana, Honduras, Mexico, Nicaragua, Panama, Paraguay, Peru, Puerto Rico, Suriname, Uruguay, Venezuela. |
| **Caribbean**: Anguilla, Antigua and Barbuda, Aruba, Bahamas, Barbados, Belize, Cayman Islands, Dominica, Grenada, Guadeloupe, Haiti, Jamaica, Martinique, , , Montserrat, Netherlands Antilles, Saint Kitts and Nevis, Saint Lucia, Saint Vincent And The Grenadines, Trinidad and Tobago, Turks and Caicos Islands, Virgin Islands, British Virgin Islands, West Indies NES. |
| **Sub-Saharan Africa**: Angola, Benin, Botswana, Burkina Faso, Burundi, Cameroon, Cape Verde, Central African Republic, Chad, Comoros, Congo, The Democratic Republic of Congo, Cote D'ivoire, Djibouti, Equatorial Guinea, Eritrea, Ethiopia, Gabon, Gambia, Ghana, Guinea, Guinea Bissau, Kenya, Lesotho, Liberia, Madagascar, Malawi, Mali, Mauritania, Mauritius, Mayotte, Mozambique, Namibia, Niger, Nigeria, Reunion, Rwanda, Sao Tome and Principe, Senegal, Seychelles, Sierra Leone, Somalia, South Africa, Swaziland, Tanzania, United Republic Of Togo, Uganda, Western Sahara, Zambia, Zimbabwe. |
| **Middle East and North Africa**: Algeria, Armenia, Azerbaijan, Bahrain, Cyprus, Egypt, Georgia, Iran, Iraq, Israel, Jordan, Kazakhstan, Kuwait, Kyrgyzstan, Lebanon, Libya, Morocco, Oman, Occupied Palestinian Territory, Qatar, Saudi Arabia, Sudan, Syria, Tajikistan, Tunisia, Turkey, Turkmenistan, United Arab Emirates, Uzbekistan, Yemen. |
| **East Asia and Pacific**: Asia NES, Brunei Darussalam, Cambodia, China, Fiji, French Polynesia, Guam, Hong Kong, Indonesia, Japan, Kiribati, Korea North, Korea South, Laos, Macau, Malaysia, Mongolia, Myanmar, Nauru, New Caledonia, Northern Mariana Islands, Palau, Papua New Guinea, Philippines, Samoa, Singapore, Solomon Islands, Taiwan, Thailand, Tibet, Tonga, Vanuatu, Viet Nam, Tuvalu, Marinas, Republic of The Marshall Islands, Federated States of Micronesia, Cook Islands, Wallis And Futuna, Ocean NES, East Timor. |
| **South Asia**: Afghanistan, Bangladesh, Bhutan, India, Maldives, Nepal, Pakistan, Sikkim, Sri Lanka. |

**Table S2. Diagnostic and procedural codes used to identify the cohort, comorbidity and outcome features**

| **Assessment** |  | **Disease or procedure** | **Period of assessment** | **ICD-10-CA codes** | **OHIP codes** |
| --- | --- | --- | --- | --- | --- |
| ***Cohort inclusion criteria applied to all maternal-child pairs*** | Fetal/infant | - Singleton, and - Liveborn, and - Survived ≥ 29 days after date of birth. | Index birth hospitalization and ≤ 12 months after birth | - Singleton status is based on NOT being a multiple gestation, as defined below by fetal/infant and maternal exclusion criteria | -- |
|  | Maternal | First obstetrical delivery ≥ 23 weeks gestation during the period of observation | Index obstetrical delivery hospitalization | MOMBABY Dataset | -- |
| ***Cohort exclusion criteria applied to all maternal-child pairs*** | Fetal/infant | Multiple gestation of 3 or more fetuses | Index birth hospitalization | O30, O31 |  |
|  | Maternal | Multiple gestation | Index obstetrical delivery hospitalization | O30, O31, Z37.2-Z37.7,  Z38.3-Z38.8 |  |
|  |  | Intrauterine fetal death/stillbirth | Index obstetrical delivery hospitalization | O36.4, P95 | -- |
| ***Main study outcome*** | Fetal/infant | Cerebral palsy | > 28 days and ≤ 48 months after date of birth | G80 – any level of diagnosis | 343 -- must be submitted by a pediatrician (OHIP Database Health Care Provider Specialty Code 26) ≥ 2 times, and ≥ 14 days between submissions |
| ***Modified study outcome excluding recognized causes of CP (for Model B)*** | Fetal/infant | *Cerebral palsy excluding an explainable cause of cerebral palsy* (as listed in the 17 segregated rows in light blue below)   - Fetus and newborn affected by placenta praevia - Fetus and newborn affected by prolapsed cord - Fetus and newborn affected by other compression of umbilical cord - Fetus and newborn affected by other and unspecified conditions of umbilical cord | Index birth hospitalization | - P02.0 - P02.4 - P02.5 - P02.6 | -- |
|  |  | Infections specific to the perinatal period | Index birth hospitalization | P35-P39 | -- |
|  |  | - Fetus and newborn affected by chorioamnionitis - Fetus and newborn affected by other abnormalities of membranes - Fetus and newborn affected by abnormality of membranes, unspecified | Index birth hospitalization | - P02.7 - P02.8 - P02.9 | -- |
|  |  | Neonatal kernicterus | Index birth hospitalization | P57 | -- |
|  |  | Fetal and neonatal hemorrhage | Index birth hospitalization | P50-P54 | -- |
|  |  | Any birth trauma diagnosed during the fetal or neonatal period | Index birth hospitalization | P10-P15 |  |
|  |  | Fetus and newborn affected by other complications of labour and delivery | Index birth hospitalization | P03 | -- |
|  |  | Fetus and newborn affected by noxious influences transmitted via placenta or breast milk | Index birth hospitalization | P04 | -- |
|  |  | Any congenital or chromosomal anomaly | Index birth hospitalization and ≤ 12 months after birth | Q00-Q99 |  |
|  |  | Meningitis or encephalitis | Index birth hospitalization and ≤ 12 months after birth | G00-G09 | -- |
|  | Maternal | Intrauterine hypoxia and birth asphyxia | Index obstetrical delivery hospitalization | P20, P21 | -- |
|  |  | Uterine rupture | Index obstetrical delivery hospitalization | O71.0, O71.1 | -- |
|  |  | Umbilical cord prolapse or vasa previa | Index obstetrical delivery hospitalization | O69 | -- |
|  |  | Amniotic fluid embolism | Index obstetrical delivery hospitalization | O88.1 | -- |
|  |  | Fetal-maternal hemorrhage | Index obstetrical delivery hospitalization | O43.0 | -- |
|  |  | Chorioamnionitis | Index obstetrical delivery hospitalization | O41.1 | -- |
| ***Modified by co-exposures (Models D-F)*** | Maternal | *Maternal placental syndromes:*   - Preeclampsia or eclampsia - Gestational hypertension - Placental abruption - Placental infarction | Index delivery hospitalization | - O13, O14, O15 - O16 - O45 - O43.1, O43.8, O43.9 | -- |
|  | Fetal/infant | Birthweight (grams) | Index birth hospitalization | -- | -- |
|  | Maternal/  infant | Gestational age at delivery/birth, in completed weeks | Index obstetrical delivery/birth hospitalization | -- |  |
|  |  |  |  |  |  |
| Covariates |  | Diabetes mellitus or gestational diabetes mellitus | ≤ 12 months before, and including, index obstetrical delivery hospitalization | E10-E14, O24 | 250 |
|  |  | Obesity | ≤ 12 months before, and including, index obstetrical delivery hospitalization | E66 | 278 |
|  |  | Tobacco use | ≤ 12 months before, and including, index obstetrical delivery hospitalization | Z72.0 | 305 |
|  | Fetal/infant or maternal | Delivery by Caesarian section | Index birth or delivery hospitalization | P03.4, O82, 74* | -- |
| Other variables | Infant | Diseases of prematurity:   - Respiratory distress syndrome - Necrotizing enterocolitis - Periventricular leukomalacia or intraventricular hemorrhage - Retinopathy of prematurity (retrolental fibroplasia) | Index birth hospitalization | - P22 - P77 - P91.2,P52 - H35.1 | -- |

**Table S3. Modified main model, also adjusting for the presence of a *maternal placental syndrome* during the index delivery hospitalization.**

|  |  | **Outcome of cerebral palsy up to age 4 years** | | |
| --- | --- | --- | --- | --- |
|  |  |  | **Hazard ratio (95% confidence interval)** | |
| **Analysis** | **Mother’s World region of origin** | **Number (rate per 1000)** | **Unadjusted** | **Adjusted**** |
| ***Comparing immigrant vs. non-immigrants*** | Non-immigrants (n = 566,668) | 1089 (1.92) | *1.00 (referent)* | *1.00 (referent)* |
|  | Immigrant (n = 177,390) | 257 (1.45) | 0.75 (0.66 to 0.86) | 0.77 (0.67 to 0.88) |
|  |  |  |  |  |
| ***Comparing immigrants by their World region vs. non-immigrants*** | Non-immigrants (n = 566,668) | 1089 (1.92) | *1.00 (referent)* | *1.00 (referent)* |
|  | Sub-Saharan Africa (n = 12,717) | 23 (1.81) | 0.94 (0.62 to 1.42) | 0.87 (0.57 to 1.32) |
|  | South Asia (n = 56,316) | 94 (1.67) | 0.87 (0.70 to 1.07) | 0.84 (0.68 to 1.05) |
|  | Caribbean (n = 10,899) | 18 (1.65) | 0.86 (0.54 to 1.37) | 0.58 (0.37 to 0.93) |
|  | Hispanic America (n = 13,417) | 22 (1.64) | 0.85 (0.56 to 1.30) | 0.83 (0.54 to 1.27) |
|  | Middle East and North Africa (n = 17,222) | 28 (1.63) | 0.85 (0.58 to 1.23) | 0.90 (0.61 to 1.32) |
|  | Western Nations and Europe (n = 27,967) | 37 (1.32) | 0.70 (0.50 to 0.96) | 0.77 (0.55 to 1.06) |
|  | East Asia and Pacific (n = 38,852) | 35 (0.90) | 0.47 (0.34 to 0.66) | 0.54 (0.39 to 0.77) |

*Adjusted for presence of a *maternal placental syndrome*, maternal age, parity, neighbourhood income quintile, any pre-pregnancy or gestational diabetes mellitus, obesity, tobacco use, Caesarean delivery, fiscal year of delivery, number of physician visits between day 1 and day 140 of pregnancy, twin pregnancy, preterm birth before 32 weeks, preterm birth from 33 to 37 weeks, small for gestational age birthweight under the 10th percentile, and large for gestational age birthweight over the 90th percentile.
