# Supplementary figures and images for: Risk of Cerebral Palsy among the Offspring of Immigrants
Source: PLoS One. 2014 Jul 14;9(7):e102275. doi: 10.1371/journal.pone.0102275 (PMC4096602; doi:10.1371/journal.pone.0102275)

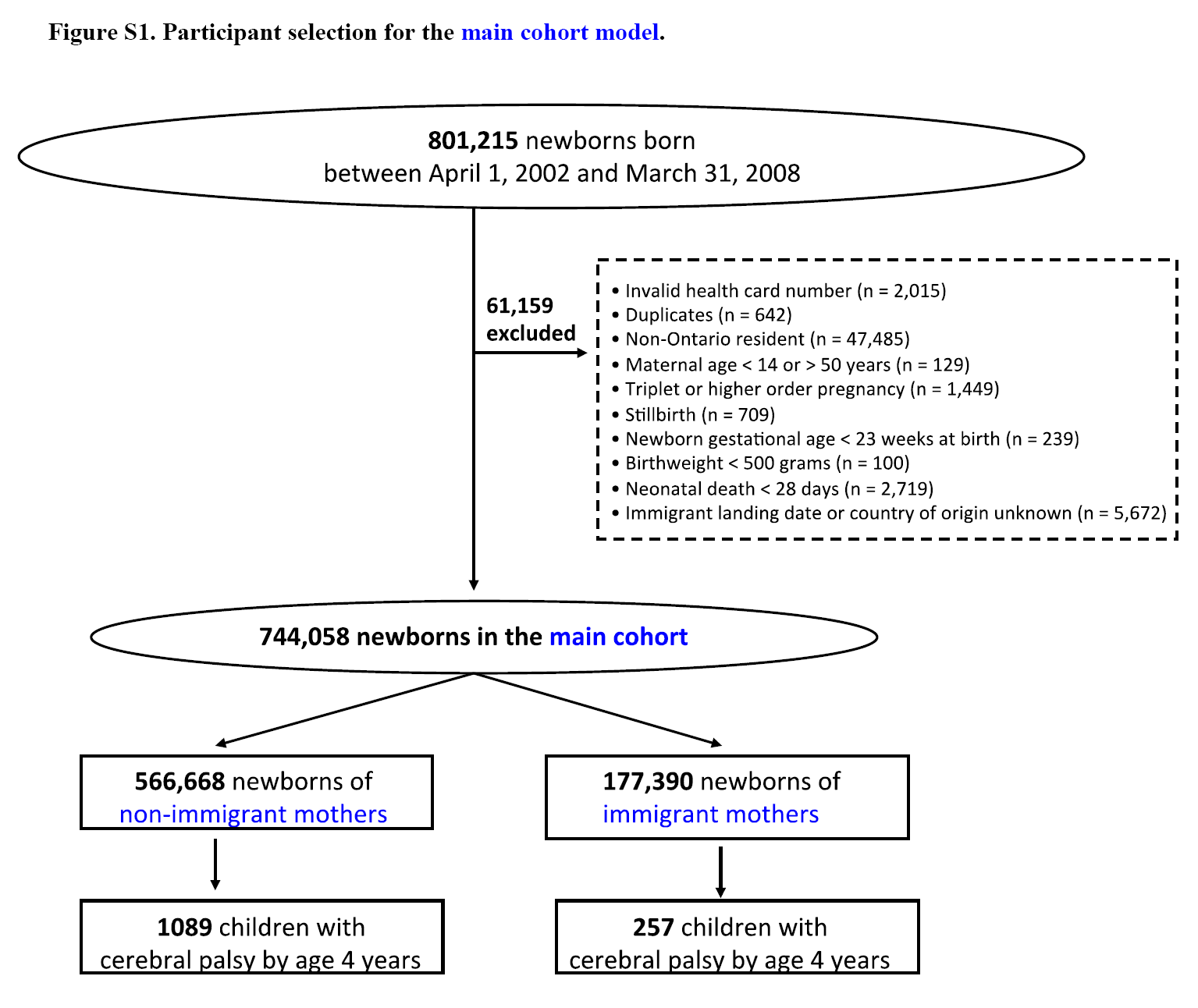

Supplement: Figure S1 — Participant selection for the main cohort model (TIF) [file pone.0102275.s002.tif]
